# Tumor mutation burden involving epigenetic regulatory genes and the *RhoA* GTPase predicts overall survival in nodal mature T-cell lymphomas

**DOI:** 10.1186/s13148-022-01395-4

**Published:** 2022-12-19

**Authors:** Luís Alberto de Pádua Covas Lage, Hebert Fabrício Culler, Guilherme Carneiro Barreto, Cadiele Oliana Reichert, Débora Levy, Renata de Oliveira Costa, Vanderson Rocha, Juliana Pereira

**Affiliations:** 1 Department of Hematology, Hemotherapy and Cell Therapy, University of São Paulo (USP), São Paulo, Brazil; 2 Laboratory of Medical Investigation in Pathogenesis and Directed Therapy in Onco-Immuno-Hematology (LIM-31), University of São Paulo (USP), Avenue Dr Enéas de Carvalho Aguiar, 155, Ambulatory Building, 1st. Floor, Room 61, Cerqueira César, São Paulo, 05403-000 Brazil; 3 Laboratory of Medical Investigation in Immunology and Histocompatibility (LIM-19), University of São Paulo (USP), São Paulo, Brazil; 4 Department of Hematology and Hemotherapy, Faculdade de Ciências Médicas de Santos (FCMS), Centro Universitário Lusíada, Santos, Brazil; 5 Fundação Pró-Sangue, Blood Bank of São Paulo, São Paulo, Brazil; 6 Churchill Hospital, Oxford University, Oxford, UK; 7 Hospital Alemão Oswaldo Cruz (HAOC), São Paulo, Brazil

**Keywords:** Nodal mature T-cell lymphomas, Epigenetics, *RhoA* mutation, Molecular biomarkers, Clinical outcomes

## Abstract

Nodal mature T-cell lymphomas (nMTCL) comprises a heterogeneous group of rare malignancies with aggressive biological behavior and poor prognosis. Epigenetic phenomena, including mutations in genes that control DNA methylation and histone deacetylation, in addition to inactivating mutations in the *RhoA* GTPase, play a central role in its pathogenesis and constitute potential new targets for therapeutic intervention. Tumor mutational burden (TMB) reflects the process of clonal evolution, predicts response to anti-cancer therapies and has emerged as a prognostic biomarker in several solid neoplasms; however, its potential prognostic impact remains unknown in nMTCL. In this study, we conducted Sanger sequencing of formalin-fixed paraffin-embedded (FFPE) diagnostic tumor samples using a target-panel to search for recurrent mutations involving the *IDH-1*/*IDH-2*, *TET-2*, *DNMT3A* and *RhoA* genes in 59 cases of nMTCL. For the first time, we demonstrated that high-TMB, defined by the presence of ≥ two mutations involving the aforementioned genes, was associated with decreased overall survival in nMTCL patients treated with CHOP-like regimens. Additionally, high-TMB was correlated with bulky disease, lower overall response rate, and higher mortality. Future studies using larger cohorts may validate our preliminary results that indicate TMB as a potential molecular biomarker associated with adverse prognosis in nMTCL.

## Introduction

Mature T-cell lymphomas (MTCL) represents a heterogeneous group of post-thymic T-cell malignant neoplasms that are highly aggressive and frequently resistant to chemotherapy based on anthracyclic agents. Patients with MTCL have an estimated 5-year overall survival (OS) around 30–40% [1, 2]. It accounts for 15% of all non-Hodgkin’s lymphomas (NHL), and according to the 2016-World Health Organization (WHO) Classification of Hematopoietic and Lymphoid Tissue Neoplasms, are categorized as predominantly nodal, extra nodal, primary cutaneous and disseminated or leukemic [1]. Although rare, these tumors show characteristic geographic distribution, being more prevalent in East Asia and South America, where it represents up to 25% of all NHL [1, 2].

The nodal subgroup (nMTCL) encompasses approximately 60–70% of MTCL cases, comprising four main histopathological variants, including peripheral T-cell lymphoma, not otherwise specified (PTCL, NOS); angioimmunoblastic T-cell lymphoma (AITL) and other nodal MTCL with T-helper follicular phenotype (nMTCL-THf), such as the follicular T-cell lymphoma with t(5;9)(q33;q22)—*ITK/SYK* rearrangement; and ALK1 (*anaplastic lymphoma kinase-1*) positive and negative systemic anaplastic large cell lymphomas (sALCL) [1]. Commonly, patients with nMTCL present unfavorable biological features at diagnosis, including inactivation of the tumor suppressor genes *TP53* and *p15INK4b/p16INK4a*, high concentration of P-glycoprotein (Pgp) into neoplastic cells and multi-drug resistance phenotype (MDR), which explain the high rates of therapeutic failure with CHOP-like (cyclophosphamide, doxorubicin, vincristine, and prednisone) regimens [2].

Epigenetic phenomena involving genes that control DNA methylation and histone deacetylation, such as *IDH-2*, *DNMT3A* and *TET-2*, play a central role in the pathogenesis of nodal MTCL, particularly in THf-phenotype subtypes [3]. This growing biological knowledge was translated into new therapeutic opportunities through the incorporation of hypomethylating agents (HMAs), such as 5-azacitide and decitabine, and histone deacetylase inhibitors (HDACi), such as vorinostat, belinostat and romidepsin into these tumor’s treatment [3–5]. Promising results from these therapies and its tolerable safety profile, with 70–75% overall response rate (ORR), either alone or in combination with CHOP-like regimens, are encouraging [4–6].

The *RhoA* gene (*Ras homolog family member A*), located on chromosome 3, encodes a small GTPase that controls the conformation of the cytoskeleton, T-cell receptor (TCR) signaling, and plays a central role in the ontogenesis of T-lymphocytes [7]. Yoo et al. [8] identified recurrent mutations involving the *RhoA* gene in nMTCL-THf patients, particularly in AITL. The *RhoA* G17V point mutation, associated with loss of function of this GTPase, is closely linked to nMTCL-THf oncogenesis, occurring in up to 60% of AITL cases [7, 8]. Currently, *RhoA* mutations are considered as diagnostic biomarkers of AITL and its correlated disorders; however, their prognostic role remains controversial [8–10].

Tumor mutation burden (TMB) has emerged as a prognostic biomarker in several solid tumors such as breast, prostate, and lung cancers [11]. High TMB has been associated with better responses to anti-cancer therapies and correlates with clinical outcomes in different malignancies. In this sense, TMB also has been considered a promising response biomarker to immune checkpoint inhibitors [12, 13]. Furthermore, TMB reflects the clonal evolution, characterized by progressive addition of molecular-genetic abnormalities, as well as intratumor heterogeneity [14]. Recently, Falchi et al. reported a correlation between high TMB on epigenetic regulatory genes and higher overall response rate (ORR) to 5-azacytidine and romidepsin in nodal MTCL [5]. However, the potential prognostic of epigenetic TMB remains largely unknown in nodal MTCL. Based on this premise, herein we aimed to establish a correlation between epigenetic TMB and prognosis in nodal MTCL, as well as searching for associations between TMB and clinical phenotype in this subgroup of neoplasms.

## Methods

We conducted a retrospective, observational and single-center study involving 59 patients with biopsy-proven diagnosis of nodal MTCL, treated at the Department of Hematology, University of São Paulo, Brazil, from January 2000 to December 2019. The study was approved by the Research Ethics Committee of our institution (number 02975012.0.0000068) and was allowed to waive the application of the Informed Consent Form. Clinical, laboratory and epidemiological data were captured from electronic medical records and Database of the NHL Group at the University of São Paulo.

All patients were tested for HIV and HTLV-1 during staging procedures, and those with positive serology were excluded. Likewise, non-systemic ALK-1 negative ALCL patients, such as those with cutaneous primary ALCL, lymphomatoid papulosis and ALCL associated with breast implants were also excluded, as well as those cases aged ≤ 18 years old. All cases underwent blood counts, biochemical tests, including kidney and liver function, serum lactate dehydrogenase (LDH) dosage, transthoracic echocardiography to estimate myocardial function pre-exposure to anthracyclines, imaging tests, including computed tomography (CT) of the neck, chest, abdomen and pelvis and/or 18-FDG-PET CT, as well as unilateral bone marrow biopsy. Selected cases underwent neuroimaging examinations, including brain magnetic resonance imaging (MRI), cerebrospinal fluid analysis, as well as endoscopic evaluation of the gastrointestinal tract.

All cases were submitted to centralized histopathological review by two experts in Hematopathology and were categorized according to the 2016-WHO Classification [1]. After morphological analysis in Hematoxylin–Eosin (HE) staining, the immunohistochemical (IHC) study was performed by a screening panel including the monoclonal antibodies Ki67 (Dako, K55, 1/1600), pan-B CD20 (Dako, L26, 1/1000), pan-T CD3 (Dako, F7.2.38, 1/500) and the marker KI-1/CD30 (cell Marque, Ber-H2, 1/1000). An extended IHC panel was applied according to the results of the HE and IHC screening study.

For cases suggestive of AITL/nMTCL-THf, IHC study incorporating the monoclonal antibodies for THf cells [CD10 (Novocastra, S6C6, 1/2000), BCL-6 (Abcam, EPR11410-43, 1/500), ICOS (Abcam, SP98, 1/100), CXCL-13 (Abcam, Ab112521, 1/300) and PD-1 (Abcam, NAT105, 1/1000)], for endothelial cells [CD31 (Dako, JC/70A, 1/100) and CD34 (Dako, QBEand-10, 1/2000)], and for follicular-dendritic cells (FDC) antigens [CD21 (Novocastra, 2G9, 1/800) and CD23 (Biocare, 1B12, 1/1000)] was conducted. In situ hybridization (ISH) for small-RNA encoded by Epstein-Barr virus using *Zyto-Fast Plus CISH implementation kit*™ (ZytoVision, Bremerhaven, Germany) was performed for all cases. Additionally, cases suspected for ALCL and PTCL, NOS were tested with the monoclonal antibodies: ALK-1 (Spring, SP8, 1/400), CD45 (Dako, 2B11 + PD7/26, 1/2000), CD15 (Dako, Carb-3, 1/50), PAX-5 (Dako, Dak-Pax5, 1/200), CD2 (Monossan, AB75m, 1/200), CD4 (Spring, SP35, 1/400), CD7 (Novocastra, CD7-272, 1/3000) and CD8 (Dako, C8/144B, 1/800). Cases with CD30 expression on neoplastic cells were stained with CD45, PAX-5 and CD15 antigens to distinguish between ALK1-negative sALCL and classical Hodgkin lymphoma (cHL).

For the molecular study, total DNA was isolated from formalin-fixed paraffin-embedded (FFPE) diagnostic tumor samples using *QIAamp DNA FFPE Tissue Kit™* (Qiagen, Hilden, Germany) after deparaffinization with xylol. Sanger sequencing with a target-panel, involving *IDH-1*, *IDH-2*, *DNMT3A*, *TET-2* and *RhoA* genes to search for recurrent mutations using *BigDye Terminator v3.1 Cycle Sequencing Kit*™ (Thermo Fisher Scientific, Wilmington, DE, USA) was performed on the thermocycler Verity 96 Well (Applied Biosystem, Foster City, CA, USA), followed by capillary electrophoresis on the 3500 Genetic Analyzer (Applied Biosystem, Foster City, CA, USA). The sequences of the primers used for target-DNA amplification were: *IDH-1* (**F**-5′CGGTCTTCAGAGAAGCCATT3′; **R**-5′GCAAAATCACATTATTGCCAAC3′), *IDH-2* (**F**-5′TGTAAAACGA CGGCCAGTAAAGAAAGAATAGTCCCTGGCTGGACCAAG3′; **R**-5′TCCCCTCTCCACCCTGG3′), *DNMT3A* (**F**-5′TTTGGTTTCCCAGTCCACTATAC3′; **R**-5′CAGGAAACAGCTATGACCAAAGAAAGAAGTGGCGGAT GACTGGCAC3′), *RhoA* (**F**-5′ TGTAAAACGACGGCCAGTAAAGAAAGAAGTTTTGTGTTTCAGCAATG G3′; **R**-5′TCTGGGAACTGGTCCTTG3′) and *TET-2* (**F**-5′TGTAAAACGACGGCCAGTAAAGAAAGAAT CAAGAACAGGAGCAGAAG3′; **R**-5′CAGGAAACAGCTATGACCAAAGAAAGAAGCATGGTTATGTATCA AGTAC3′; **F**-5′TGTAAAACGACGGCCAGTAAAGAAAGAACTGTCTCTGGCTGACAAAC3′; **R**-5′CAGGAA ACAGCTATGACCAAAGAAAGAACTTCATTCAAGGCACACCG3′; **F**-5′TGTAAAACGACGGCCAGTAAA GAAAGAAAACACAGAGCACCAGAGTG3′; **R**-5′CAGGAAACAGCTATGACCAAAGAAAGAATGCATGTT GTGCAAGTCTC3′). qRT-PCR reactions of five healthy FFPE lymph node samples were carried out in parallel as a control. The sequences obtained were analyzed using *Bioedit* and *Sequencing Analysis v5.*4 softwares. Mutations were categorized and annotated using the VEP (*Ensembl Variant Effector Predictor*) and PolyPhen (*Polymorphism Phenotyping*) online tools, as well as the COSMIC (*Catalog of Somatic Mutations in Cancer*) platform.

For statistical analysis, only non-synonymous mutations were considered. Survival curves were constructed using the Kaplan–Meier method and the Log-Rank test was used to establish correlation between TMB, overall survival (OS) and progression-free survival (PFS). Chi-square test was used to establish a relationship between TMB or recurrent mutations and clinical-laboratorial characteristics. Statistical tests were performed using the software STATA 12.0 and a *p* value < 0.10 was considered statistically significant.

## Results

The median age of all cohort was 50 years (IqR: 38–61) and 57.6% (34/59) were male. Thirty-seven percent (22/59) were classified as systemic ALK1-negative ALCL, 27.1% (16/59) as PTCL, NOS, 20.3% (12/59) as ALK1-positive ALCL and 15.2% (9/59) as AITL/nMTCL-THf phenotype. Thirty-nine percent (23/59) had bulky disease ≥ 7 cm, 83% (49/59) had B-symptoms, 94.9% (56/59) had advanced clinical stage—Ann Arbor/Cotswolds III/IV, 27.1% (16/59) had *Eastern Cooperative Oncology Group* scale (ECOG) ≥ 2, 27.1% (16/59) had ≥ 2 extranodal sites involved by NHL, and 55.9% (33/59) had *International Prognostic Index* (IPI) ≥ 3. Bone marrow infiltration was detected in 11.9% (7/59) of cases, and the median of lactic dehydrogenase (LDH) level was 488 UI/L (IqR: 297–737). Seventy-six percent (45/59) received up-front therapy with anthracycline-based chemotherapy (up to 6–8 cycles of CHOP-21 or CHOEP-21 regimens), 23.7% (14/59) experienced radiotherapy, and 33.3% (20/59) were consolidated with autologous stem-cell transplantation (ASCT).

With a median follow-up of 3.7 years (95% CI 0.9–12.4), the estimated 2-year OS and PFS were 57.1% (95% CI 45.5–70.4) and 49.2% (95% CI 34.0–59.2), respectively. The estimated 2-year OS was 72.7% (95% CI 49.1–86.7) for ALK1-positive ALCL, 58.3% (95% CI 27.0–80.1) for ALK1-negative sALCL, 49.2% (95% CI 23.5–70.6) for PTCL, NOS, and 44.4% (95% CI 13.5–71.9%) for AITL/nMTCL-THf. Similarly, the estimated 2-year PFS was 68.2% (95% CI 44.6–83.4) for ALK1-positive ALCL, 58.3% (95% CI 27.0–80.1) for ALK1-negative sALCL, 12.5% (95% CI 2.0–32.8) for PTCL, NOS, and 22.2% (95% CI 3.3–51.3%) for AITL/nMTCL-THf. The ORR after up-front therapy was 59.3% (95% CI 45.7–71.9), 54.2% (32/59) reached complete remission (CR) and 5.1% (3/59) partial response (PR). The mortality rate during all follow-up was 52.5% (95% CI 39.1–65.7), and the main cause of death was progression of disease, encompassing 48.3% (15/31) of the deaths.

**Fig. 1:**
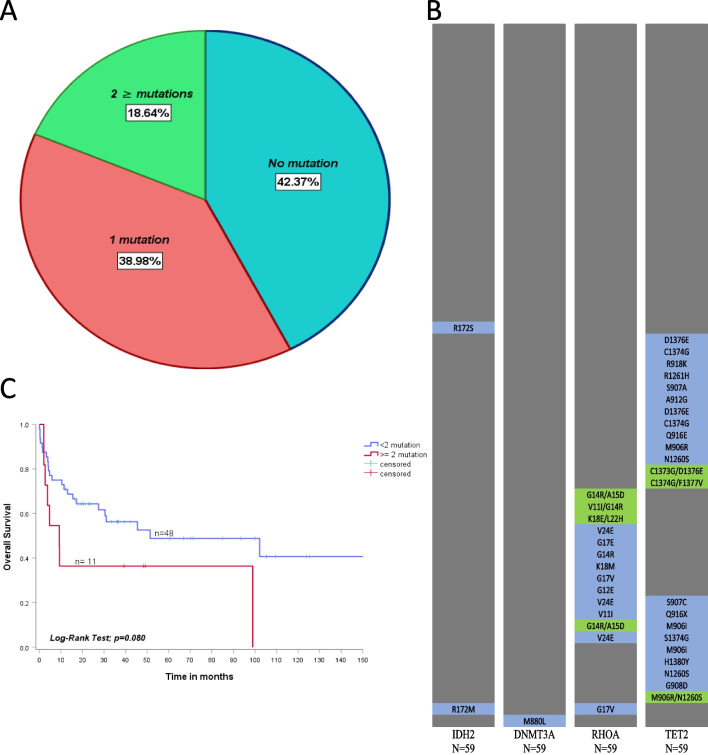
**A** TMB stratified by the absence of mutations (42.4% of cases), presence of 1 mutation (38.9%), and two or more mutations (18.6%); **B** Heatmap demonstrating mutations detected in *IDH-2*, *DNMT3A*, *RhoA* and *TET-2* genes across 59-Brazilian patients with nMTCL; **C** OS curves of 59-Brazilian patients with nodal MTCL stratified according epigenetic TMB—Blue-line represents low-TMB patients (< 2 mutation) and red-line represents high-TMB cases (≥ 2 mutation)

For the whole cohort of nodal MTCL (*n* = 59), missense or non-sense mutations on *IDH-1* gene were not observed in any case (0%–0/59), *IDH-2* mutations occurred in 3.3% (2/59) of the cases, *DNMT3A* in 1.6% (1/59), *RhoA* in 23.7% (14/59) and *TET-2* in 37.2% (22/59). Mutations involving the *IDH-2* gene included R172S and R172M; *DNMT3A* mutation included M880L. The *RhoA* gene revealed the mutations K18E, L22H, G14R, K18M, G12E, G17V, V24E, A15D, V11I and G17E. *TET-2* mutations included A912G, C1374G, D1376E, S907C, F1377V, Q916X, M906I, S1374G, R918K, R1261H, S907A, N1260S, Q916E, M906R and H1380Y (Fig. 1B).

Among the 59 patients included, 25/59 (42.4%) had no mutations in these target-genes, 23/59 (38.9%) had 1 mutation, and 11/59 (18.6%) had two or more mutations (Fig. 1A). *TET-2* mutations were homogeneously distributed among the different histopathological subtypes of nodal MTCL (*p* = 0.99), however *RhoA* mutations were recurrent in non-ALCL subtypes (PTCL, NOS and AITL), particularly in those cases with THf-phenotype (*p* = 0.06), characterized by expression of at least two THf-antigens, including CD10, BCL-6, CXCL-13, ICOS and PD-1. Among cases with ≥ 2 mutations, the association *RhoA*-mut/*TET-2*-mut was commonly observed. Out of the 14 *RhoA*-mutated cases, six (42.8%) also had *TET-2* mutations, suggesting a biological cooperation for the nMTCL-THf cases lymphomagenesis.

The cases were categorized into low TMB (< 2 mutations involving epigenetic regulatory genes and the *RhoA* GTPase) [*n* = 48/59–81.3%] and high TMB (≥ 2 mutations involving the same tested genes) [*n* = 11/59–18.7%]. The median OS was 51.4 months (95% CI 0–124.1) for low TMB and only 3.1 months (95% CI 3.1–15.2) for high TMB, *p* = 0.08. The estimated 2-year OS was 64.3% (95% CI 50.6–78.0) for low TMB and 36.4% (95% CI 8.0–65.0) for high TMB (Fig. 1C). Similarly, the median PFS was 13.3 months (95% CI 0–29.4) for low TMB and 6.3 months (95% CI 0.9–11.6) for high TMB, *p* = 0.139. The estimated 2-year PFS was 46.1% (95% CI 20.0–72.1) for low TMB and 25.0% (95% CI 17.5–67.5) for high TMB.

As summarized in Table 1, high TMB was statistically significantly associated with adverse clinical-biological characteristics and more aggressive clinical phenotype in patients with nodal MTCL, including male gender (81.8% for high TMB vs. 52.1% for low TMB, *p* = 0.06), high tumor volume represented by bulky disease ≥ 7 cm (90.9% for high TMB vs. 54.2% for low TMB, *p* = 0.01), decreased ORR to CHOP-like chemotherapy (36.4% for high TMB vs. 60.4% for low TMB, *p* = 0.08), and higher mortality (81.8% for high TMB vs. 47.9% for low TMB, *p* = 0.04). However, there was no statistically significant association between TMB and International Prognostic Index (IPI)/Prognostic Index for T-cell Lymphomas (PIT), performance status by ECOG scale, B-symptoms, Ann Arbor/Cotswolds clinical stage, bone marrow infiltration, and number of extra nodal sites affected by NHL.

**Table 1:** Correlation between TMB and clinical phenotype for 59-Brazilian patients with nMTCL

Characteristic	Low-TMB (*N*, %) (48/59–81.3%)	High-TMB (*N*, %) (11/59–18.7%)	*p* value
Male gender	25 (52.1%)	09 (81.8%)	*p* = 0.06
IPI ≥ 3 points	26 (54.2%)	07 (63.6%)	*p* = 0.56
PIT ≥ 3 points	23 (47.9%)	07 (63.6%)	*p* = 0.34
ECOG ≥ 2	25 (52.1%)	08 (72.8%)	*p* = 0.21
Bulky disease ≥ 7 cm	26 (54.2%)	10 (90.9%)	*p* = 0.01
B-symptoms	39 (81.3%)	10 (90.9%)	*p* = 0.41
BM infiltration	05 (10.4%)	02 (18.2%)	*p* = 0.78
≥ 2 extra nodal sites	13 (27.1%)	03 (27.3%)	*p* = 0.87
Clinical stage III/IV	45 (93.8%)	11 (100%)	*p* = 0.75
ORR	29 (60.4%)	04 (36.4%)	*p* = 0.08
Mortality	23 (47.9%)	09 (81.8%)	*p* = 0.04

*TMB* tumor mutation burden, *IPI* international prognostic index, *PIT* prognostic index for peripheral T-cell lymphomas, *ECOG* Eastern Cooperative Oncology Group, *BM* bone marrow, *ORR* overall mortality rate

## Discussion

In this study, we demonstrated for the first time that high-TMB, involving epigenetic regulatory genes and the *RhoA* GTPase, adversely affected the OS of patients with nodal MTCL. In our cohort, patients with ≥ 2 mutations involving the *IDH-1*/*IDH-2*, *DNMT3A*, *TET-2* and/or *RhoA* genes, accessed by Sanger sequencing in diagnostic FFPE biopsies, had an estimated 2-year OS of only 36.4% versus 64.3% for cases with less than 2 mutations, *p* = 0.08. Similarly, we were able to establish an association between a high number of mutations involving these epigenetic modifiers and unfavorable clinical phenotype in nodal MTCL. Patients with high-TMB showed statistically significantly higher tumor volume, represented by bulky disease ≥ 7 cm (*p* = 0.01), as well as higher mortality (*p* = 0.04) and elevated failure rate to anthracycline-based chemotherapy (*p* = 0.08) compared to those cases with low-TMB.

Due to the frequent overlapping of clinical, pathological, phenotypic and molecular-genetic findings among the four main histological subtypes of nodal MTCL recognized by the latest WHO Classification of Hematopoietic Neoplasms (WHO-2016) and taking into account the high rate of therapeutic failure with conventional chemotherapy regimens, the discovery of new diagnostic and prognostic biomarkers is fundamental [2, 15]. In this sense, our group recently demonstrated that peripheral monocyte count ≥ 1.5 × 10^9^/L at diagnosis, overexpression of the *CCNA2* gene and CHEK1 protein, high tissue expression of the *GATA-3* gene and mutations involving the *TET-2* gene may be potential biomarkers capable of assisting in the diagnostic discrimination of the different nodal MTCL histological subtypes. These biomarkers were also capable of predicting adverse clinical outcomes for MTCL [16–19].

Such new genetic-molecular biomarkers may also represent specific targets for therapeutic intervention. The high concentration of *P*-glycoprotein (Pgp) in MTCL tumor cells, as well as the frequent inactivation of tumor suppressor genes, such as *TP53* and *p15INK4b*/*p16INK4a*, confer a multidrug resistance phenotype (MDR), thus explaining the poor results achieved with regimens based on anthracyclic agents and vinca alkaloids in the up-front therapy of nodal MTCL [2, 20–23]. Aiming to improve the therapeutic results, new target-drugs have been considered for the management of nodal MTCL, specifically for cases with follicular T-helper (THf) origin. In this sense, the incorporation of the anti-CD30 immunoconjugate brentuximab vedotin and the use of epigenetic modifiers based regimens, such as the association CHOP plus 5-azacytidine or the combination of 5-azacytidine plus romidepsin, have been shown to be safe and effective in the management of ALCL and nMTCL-THf, respectively [5, 6, 24, 25].

Several studies firstly conducted in myeloid neoplasms, such as myelodysplastic syndromes (MDS) and acute myeloid leukemias (AML), appointed to the recurrence of *TET-2* mutations in these tumors and correlate such mutations with an adverse phenotype, as well as with responsiveness to HMAs [26–29]. *TET-2* mutations, together with other mutations involving epigenetic regulatory genes also play a central role in the oncogenesis of nodal MTCL-THf, and different groups associate their occurrence with greater biological aggressiveness and poor outcomes in these tumors [19, 30].

Based on this biological knowledge, Falchi et al. [5] conducted a phase 2 trial involving 25 patients with nodal MTCL up-front treated with the epigenetic modifiers 5-azacytidine and romidepsin. In that study, tumor samples were submitted to next-generation sequencing (NGS) to search for mutations involving epigenetic regulators. The authors demonstrated that responders, particularly those with the THf-phenotype, had a greater mutational burden in genes involved in DNA methylation and histone deacetylation, suggesting that high TMB involving epigenetic modifiers may be a predictor of therapeutic response to HMAs and HDACi in nodal MTCL [5]. However, due to the small sample size (*N* = 25), these authors were unable to assess the potential prognostic impact of epigenomic TMB on nodal MTCL, different to what was demonstrated by our group. On the other hand, as our cohort was primarily treated with CHOP-like regimens, we could not test association between the number of mutations involving epigenetic regulators and responsiveness to HMAs and HDACi.

Similarly to Falchi et al. [5], other research groups were also able to establish an association between high TMB and therapeutic susceptibility to different anti-cancer agents in solid cancers. Studies involving patients with prostate, breast, lung, pancreas tumors and high-grade gliomas demonstrate the ability of high TMB to predict response to immunotherapy, including PD-1/PD-L1 inhibitors [31–33]. Recently, the immune-checkpoint inhibitor pembrolizumab was approved by the FDA for the treatment of adults and children with advanced cancers, who have a high TMB, defined as ≥ 10 mutations/megabase of tumor DNA after failure of other chemotherapeutic agents.

A limitation related to our study refers to the restricted gene panel tested. Next-generation sequencing with broader gene panels, particularly whole-exome sequencing (WES), might be able to more reliably estimate the TMB, through genome-wide scanning and the construction of an index contemplating the number of mutations found per DNA mega-bases (mut/Mb). Although we used the Sanger technique, our panel incorporated the main regulators involved in the biology of nodal MTCL and included primers designed to access the main recurrent mutations described in these malignancies [8, 34–36].

## Conclusion

In conclusion, to our knowledge, these results showed for the first time that high TMB, defined here by the presence of two or more mutations involving epigenetic regulatory genes and the *RhoA* GTPase, was associated with decreased OS in patients with nodal MTCL. High epigenetic TMB was also correlated with adverse parameters, such as bulky disease, decreased ORR for CHOP-like regimens and higher mortality, and may represent a predictor of response to HMA and HDACi, as previously suggested by other groups. Although preliminary and lacking external validation, these data highlight the potential of epigenetic/GTPase TMB as an important prognostic biomarker in nodal MTCL.

## Data Availability

All data generated and analyzed during this study are included in this published article. The raw data for this study are in possession of the correspondence author and may be fully available in the event of a request to the correspondence author via e-mail.
